# Multiple Chromosomal Rearrangements Structured the Ancestral Vertebrate *Hox*-Bearing Protochromosomes

**DOI:** 10.1371/journal.pgen.1000349

**Published:** 2009-01-23

**Authors:** Vincent J. Lynch, Günter P. Wagner

**Affiliations:** Department of Ecology and Evolutionary Biology, Yale University, New Haven, Connecticut, United States of America; National Institute of Genetics, Japan

## Abstract

While the proposal that large-scale genome expansions occurred early in vertebrate evolution is widely accepted, the exact mechanisms of the expansion—such as a single or multiple rounds of whole genome duplication, bloc chromosome duplications, large-scale individual gene duplications, or some combination of these—is unclear. Gene families with a single invertebrate member but four vertebrate members, such as the *Hox* clusters, provided early support for Ohno's hypothesis that two rounds of genome duplication (the 2R-model) occurred in the stem lineage of extant vertebrates. However, despite extensive study, the duplication history of the *Hox* clusters has remained unclear, calling into question its usefulness in resolving the role of large-scale gene or genome duplications in early vertebrates. Here, we present a phylogenetic analysis of the vertebrate *Hox* clusters and several linked genes (the *Hox* “paralogon”) and show that different phylogenies are obtained for *Dlx* and *Col* genes than for *Hox* and *ErbB* genes. We show that these results are robust to errors in phylogenetic inference and suggest that these competing phylogenies can be resolved if two chromosomal crossover events occurred in the ancestral vertebrate. These results resolve conflicting data on the order of *Hox* gene duplications and the role of genome duplication in vertebrate evolution and suggest that a period of genome reorganization occurred after genome duplications in early vertebrates.

## Author Summary

The genome of vertebrates has expanded greatly in gene number since our last common ancestor with invertebrates. While it is clear that genome expansions occurred early in the evolution of vertebrates, the mechanisms of that expansion—such as a single or multiple rounds of whole genome duplication, chromosome duplications, large-scale individual gene duplications, or some combination of these—is unclear. Central to this debate has been the duplication history of *Hox* clusters, which ancestrally have four copies in vertebrates, but only a single copy in invertebrates. This 1∶4 ratio has been used to support the hypothesis that two rounds of whole-genome duplications occurred in early vertebrates (named the 2R model); however, the phylogeny of the *Hox* clusters and its linked genes (the *Hox* paralogon) seem to contradict this model. Here, we use phylogenetic methods to infer that two chromosomal rearrangements occurred shortly after the genome duplications within the *Hox* paralogon. These results resolve the apparent conflict between the duplication order of the *Hox* paralogon and the 2R model and suggest that vertebrates are pseudo-octoploids.

## Introduction

**Figure 1:**
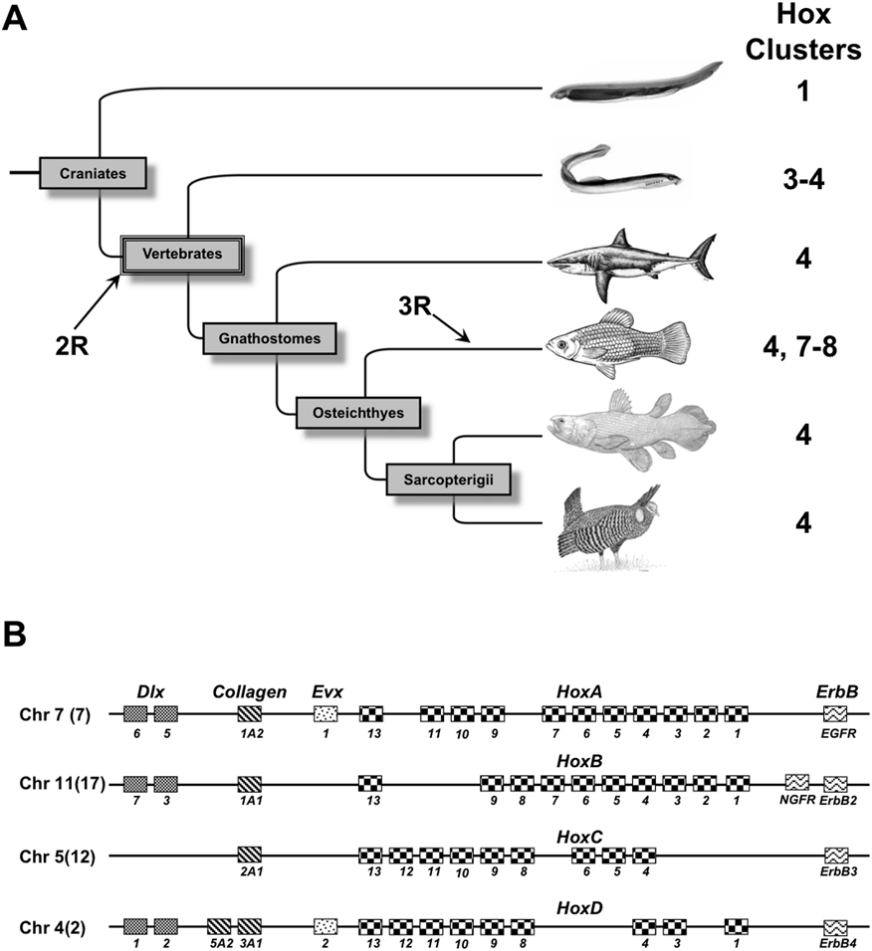
A, The phylogeny of Craniates showing the phylogenetic locations of genome duplications. Note there was an additional genome duplication in fish (3R). The number of *Hox* clusters in each group is shown on the right. B, Schematic representation of the “core” *Hox* paralogon in the ancestral vertebrate. The chromosomal location (Chr #) of the paralogons in the inferred ancestral vertebrate are shown. Gene family names are given above, and gene names are given below each cartoon.

Ohno's hypothesis [1] that two-rounds of whole genome duplication (the 2R-model) occurred in the ancestor of extant vertebrates, over 450 million years ago (Figure 1A), has generally gained wide acceptance. Recently, however, the mechanisms of that genome expansion have been debated, with some studies finding strong support for two-rounds of whole genome duplication [2]–[7] while others have found support for a single round but not two rounds of whole genome duplication [8]–[10]; some authors have even questioned whether there is any support for whole genome duplications in the evolution of vertebrates [11]–[16]. Thus, even though there is wide support for 2R-model, the evidence for it is still conflicting. Central to this debate has been the duplication history of *Hox* clusters and associated linked genes (the “*Hox* paralogon”, Figure 1A/B). Although the chromosomal order of genes within the *Hox* paralogon vary within living gnathostomes [17]–[19], ancestrally the four vertebrate *Hox* clusters (*HoxA-D*) were closely linked to at least three other gene families such as *Dlx*, *Col and ErbB*. However, there is only a single cluster with associated linked genes in invertebrates [20]. This 1∶4 ratio has been used to support the most widely held version of the 2R-model in which two rounds of whole genome duplications were followed by extensive gene loss.

Hughes [14] has argued that gene families can be used to support two-rounds of whole genome duplication only if: 1) the “extra” vertebrate genes duplicated on the vertebrate stem-lineage and thus are vertebrate-specific paralogs, and 2) gene phylogenies show a symmetrical ((A,B)(C,D)) topology indicating two duplication events. Using these criteria, Hughes [14], Friedman and Hughes [11],[12] and Hughes, da Silva and Friedman [16] surveyed the duplication history of developmentally important genes, the *Hox* clusters and genes within the Hox paralogon (among others) and found that the majority of duplication orders were inconsistent with the ((A,B)(C,D)) topology, including the *Hox* clusters themselves, violating assumption 2. These authors concluded that there was little support for the 2R hypothesis and that genome duplications did not structure the *Hox*-bearing chromosomes, respectively (although see [21]).

The phylogenetic analyses of Hughes et al. [16] have been criticized on several grounds, particularly incomplete taxon and gene sampling [2]. In a detailed reanalysis of Hughes et al's data, Larhammar et al. [2] found that the majority of genes in the analysis were either recent tandem duplications or had multiple paralogs with complex translocation histories that made them unsuitable for inferring support of the 2R hypothesis. Of the remaining 20 families, the duplication history of 14 were consistent with 2R (for example *ITGB*, *NHR* and *ACCN*), while only 6 (*AQP*, *ErbB*, *GLI*, *GNB*, *ITGA*, *NOS* and *SCN*) had phylogenies that differed from the *Hox* clusters and contradict the 2R model. Larhammar et al. [2] concluded that available data were consistent with block/chromosome duplications and that the duplications likely occurred so rapidly that phylogenetic signal did not have time to accumulate, leading to the conflicting and poorly resolved gene phylogenies often cited as evidence against 2R.

Seriously confusing the debate is the duplication history of the *Hox* clusters themselves and the gene families most closely linked to the clusters (the “core” *Hox* paralogon), which have duplication histories that are apparently different from each other and the *Hox* clusters. For example, based on minimizing the cost of gene losses after cluster duplications, Kappen and Ruddle [22] found a single best tree with the order ((A,B)(C,D)), however, the next best tree with the order (B,(A,(C,D))) was only a single step away. The (B,(A,(C,D))) topology was also found by Zhang and Nei [23] using distance methods, but these authors could not reject an ((A,B)(C,D)) topology because internal branch support was low. The most detailed study of Hox cluster duplications and the duplication of the *Hox*-linked collagen (*Col*) genes found that likelihood, parsimony and distance phylogenetic inference methods and minimum-evolution branch length tests converged on the ((A,B)(C,D)) topology, but favored placing the root at the HoxD cluster leading to the duplication order (D(A(B,C))) [24].

Although there are only three paralog groups of the *Dlx* genes in most vertebrates, invertebrates have only a single group so the duplication history of *Dlx* genes can still contribute to the debate on which two clusters are most closely related. A detailed analysis of *Dlx* genes from multiple vertebrates, including shark, lamprey and invertebrates was consistent with a (D(B,A)) duplication order [25],[26]. While this topology cannot address the relationship of these genes with respect to the *HoxC* cluster, it suggests that clusters *B* and *A* are more closely related to each other than *HoxD* and a (D,C,(B,A)) topology.

At the other end of the paralogon (Figure 1), Hughes et al. [16] found that the duplication order of the *ErbB* genes strongly supported the (D(C(B,A))) topology, a duplication order not previously found in any previous analysis. Close examination of this data, however, indicates that the chromosomal location of *HoxC/ErbB4* and *HoxD/ErbB3* clusters was mislabeled (see Hughes et al. [16] Figure 4) and therefore the phylogeny of *ErbB* genes with respect to the *Hox* clusters was incorrect. The corrected topology of *ErbB* with respect to the *Hox* clusters is (C(D(B,A)). Again, a topology not previously supported.

Given these multiple conflicting topologies, it is clear that inferring the duplication history of *Hox* clusters and closely linked genes is extremely complicated. To clarify potential reasons for this uncertainty and resolve the duplication history of this region, we conducted a phylogenetic analysis of a core set of genes in the *Hox* paralogon (*Dlx*, *Col*, *Hox and ErbB*) that are closely linked and have no evidence of translocation to other chromosomes since the diversification of extant vertebrates. Our analysis indicates that there are two competing phylogenies that divide the members of the core *Hox* paralogon; *Dlx* and *Col* share a (D(C(A,B))) topology while *Hox* and *ErbB* share a (B(A(C,D))) topology. We suggest that these competing phylogenies can be resolved if two chromosomal rearrangements occurred after the clusters duplicated but before the diversification of extant vertebrates. Indeed, this scenario has been suggested to resolve incongruent branch orders of linked genes and supports the hypothesis that the ancestral vertebrate may have been pseudo-octoploid [18].

## Results

We used several methods of phylogenetic inference because there are strengths and limitations to each method [27]. For example, neighbor-joining (NJ) and minimum evolution (ME) are widely-used, fast and perform well when divergence between sequences is low (like all methods), but a potentially serious limitation for these distance methods is that the inferred distances between genes may not accurately reflect the actual evolutionary distances between them. While compensating for variation in divergence rates can correct the inferred distances, as the degree of variation and divergence increase the effectiveness of corrections decrease [27]. Thus, when trying to infer older relationships, distance methods can fail or lead to strongly supported but incorrect results. Bayesian inference (BI) and maximum likelihood (ML) methods overcome these limitations by being based on an explicit model of nucleotide substitution that accounts for variation in evolutionary rates between nucleotide sites, but differ in how branch supports are assessed [27]. Like most other phylogenetic methods, ML use nonparametric bootstrapping to generate a confidence limit on branch supports. While widely used, bootstrap support values are generally conservative and underestimate true support when the signal-to-noise ratios are low [27]. On the other hand, BI uses the posterior distribution of trees sampled during the tree search to indicate branch support and reflect the probability the branch is correctly inferred given the data and the model; posterior probabilities generated from BI more accurately reflect branch support, but can be prone to over estimate confidence in clade support [27]. By utilizing multiple phylogenetic methods we can assess the impact of each methods assumptions on the resulting phylogeny, in addition congruence in the inferred topology between multiple methods can itself be taken as support for the topology [27].

Our phylogenetic analysis included: (1) *HoxA-D* clusters from human (*Homo sapiens*), chicken (*Gallus gallus*), frog (*Xenopus tropicalis*), coelacanth (*Latimeria chalumnae*), shark (*Heterodontus francisci*) *HoxA and HoxD* clusters and the single *Amphioxus Hox* cluster; (2) *Col1A2*, *Col2A1*, *Col1A1* and *Col3A1* genes from human (*Homo sapiens*), mouse (*Mus musculus*), dog (*Canis familiaris*), cow (*Bos taurus*) and chicken (*Gallus gallus*), *Col2A1* and *Col3A1* from frog (*Xenopus tropicalis*), and the single *Collagen* gene from Amphioxus; (3) *Dlx1/2*, *Dlx6/5* and *Dlx4/3* genes from human (*Homo sapiens*), mouse (*Mus musculus*), shark (*Heterodontus francisci*) and the single *Dlx* gene pair from *Amphioxus*, *Saccolossus* and *Ptychodera*; and (4) *EGFR*, *ErbB2*, *ErbB3*, and *ErbB4* genes from human (*Homo sapiens*), mouse (*Mus musculus*), rat (*Rattus norvegicus*), dog (*Canis familiaris*), opossum (*Monodelphis domestica*), frog (*Xenopus leavis*) and the single *ErbB* gene of *Ciona intestinalis*. Teleost (bony fish) genes were not included because of the additional genome duplication in the stem-lineage of euteleosts.

**Figure 2:**
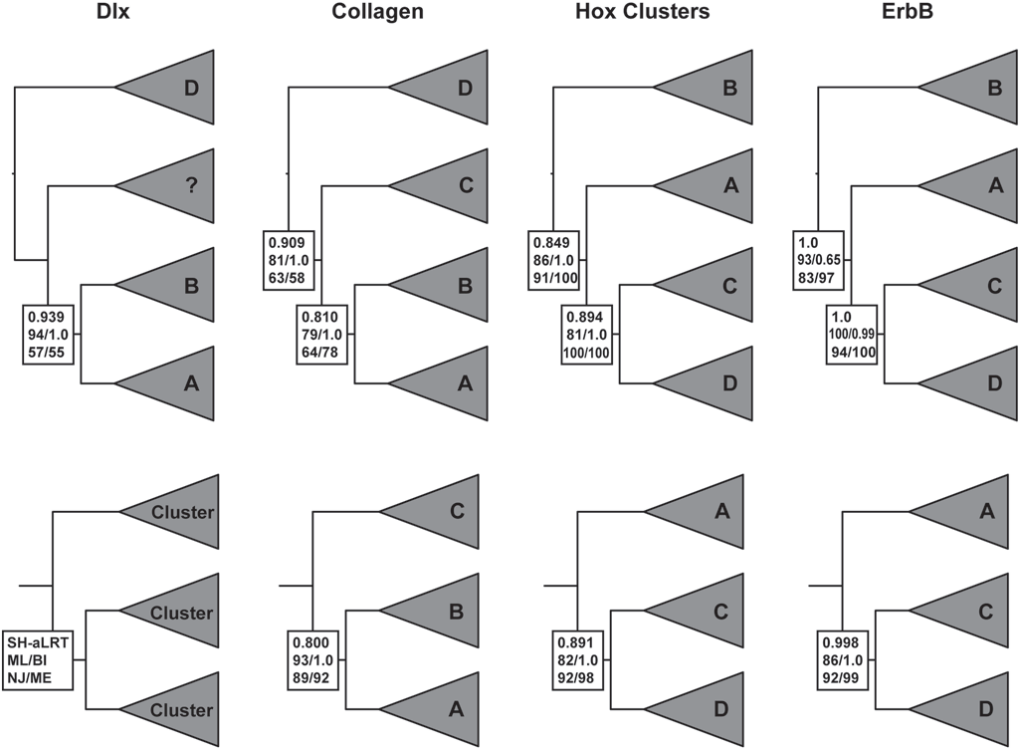
Gene trees for the “core” *Hox* paralogon. Gene names are shown with respect to which *Hox* cluster they are linked to, for example *Col3A1* is linked to the *HoxD* cluster and is shown as “D” while *Col1A2* is linked to *HoxA* is and shown as “A” (see Figure 1). Branch support values are shown for internal branches with the SH-like approximate likelihood ratio test results shown upper (HS-aLRT), maximum likelihood(ML)/Bayesian inference(BI) shown middle, and neighbor-joining(NJ)/minimum evolution(ME) shown lower (see inset). Full taxon trees are shown in the upper row, while the lower row shows the topology and branch support values when the most basal gene cluster excluded and trees re-inferred.

Phylogenetic analyses of the *Hox*, *Col*, *Dlx* and *ErbB* genes were performed using Bayesian inference (BI), maximum likelihood (ML), neighbor-joining (NJ) and minimum evolution (ME). Each method found the same topology for each gene with strong to moderate support (Figure 2). The most striking feature of the phylogenetic analyses was the split in inferred topologies between *Dlx/Col* and *Hox/ErbB*. *Col* genes support and *Dlx* genes are consistent with a (D(C(B,A))) duplication order while the *Hox* clusters and *ErbB* genes support a (B(A(C,D))) topology. Interestingly, the only difference between these two topologies is the location of the root; all genes converge on the unrooted topology (A,B(C,D)).

Several authors have noted that symmetrical ((A,B)(C,D)) topologies can be inferred as sequential (A(B(C,D))) because of long branch attraction, for example, if the out-group and one in-group clade evolve particularly fast [28]. This “pull of the past” artifact causes the most rapidly evolving in-group to cluster with the out-group solely because of homoplasy; when rooted by the out-group the resulting trees will not longer be symmetric [28]. Although problems of long-branch attraction (LBA) may be overrated [29] and do not appear to be effecting this data (see below), it is still a serious concern when genes that supposedly share a duplication history are inferred to have different phylogenies. One simple test is to remove the most basally placed in-group and re-infer the trees [29]. If the new in-group branching order is the same as the full dataset then long-branch attraction is unlikely to cause the sequential topology. Applying this test to the *Col*, *Hox* and *ErbB* data does not change the inferred branching order (Figure 2), thus long-branch attraction is unlikely to cause misplacement of the root. (*Dlx* was excluded from this analysis because there are only 3 vertebrate *Dlx* paralogs.)

A potential problem with phylogenetic inference of lineages that split rapidly is that short branches can contain little phylogenetic signal and much noise [28]. This unfavorable signal-to-noise ratio can lead to erroneous tree inferences that are essentially rooted randomly but with strong support [28]. Similarly, rooting trees using an out-group has been shown to produce incorrect trees when the in-group internal branch lengths are short [28]. We examined the effect of branch lengths on tree topology by simulating datasets with a (B(A(C,D))) topology and increasing internal branch lengths. These simulated datasets were used for ML and NJ tree inference to find the internal branch-length at which tree support collapses or becomes misleading.

**Figure 3:**
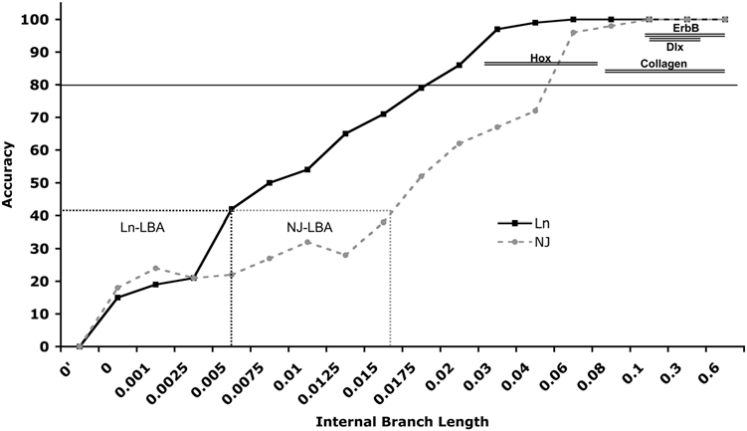
Accuracy plot. Results from the simulation study analyzing the effects of branch length on accuracy of tree inference. The internal branch length of the simulated data (X-axis) is plotted against the accuracy (Y-axis) of the inferred trees. The branch lengths that show strong long-branch attraction (LBA) biases are boxed (dashed-line) and labeled. The range of internal branch lengths for each gene family is plotted against the average internal branch support for that family obtained from phylogenetic analyses of real data shown in Figure 2 (shown as double lines). Note that branch lengths for each gene are well outside the range expected to be influenced by LBA and support is greater than 80% of bootstrap replicates (solid black line).

The results of the branch-length simulations (Figure 3) indicate that at extremely short internal branch lengths (0.001–0.0025), the root is consistently misplaced at the base of the C clade (∼43/100), which is the longest branch in both the real and simulated data (Table 1) along with the outgroup, indicating misplacement results from long branch attraction. At short internal branch lengths (0.005–0.015) the root is placed at C less often, but still at high frequency (∼28/100). At moderately short branch lengths (0.0175–0.02), however, there is a dramatic decrease in the frequency of trees rooted at C (∼5/100) indicating little detrimental effects from LBA. Above branch lengths of 0.025 no trees are misrooted at C and the majority are rooted correctly indicating no LBA artifacts. Thus, branches of length >0.0175 should be free, or nearly so, of long-branch attraction artifacts and other errors associated with random rooting at short internal branches. Indeed, the length of the internal branches (B(A,C,D) and (B,A(CD)) for *Dlx*, *Col*, *Hox* and *ErbB* genes are significantly greater than 0.0175 (Table 1), indicating the LBA and misrooting are unlikely causes of the divergent rootings.

**Table 1:** Branch length data for the stem of *Dlx*, *Col*, *Hox* and *ErbB* clusters.

Gene	Branch length	SD
*Dlx*		
*6/5*	0.170	0.043
*4/3*	0.203	0.051
***1/2***	***0.414***	***0.061***
*Collagen*		
*1A2*	0.347	0.061
*1A1*	0.157	0.047
*2A1*	0.094	0.026
***3A1***	***0.499***	***0.037***
Int.	0.077	0.013
*Hox*		
*A*	0.029	0.011
***B***	**0.055**	**0.013**
*C*	*0.096*	*0.011*
*D*	0.050	0.015
Int.	0.036	0.017
*ErbB*		
*EGFR*	0.127	0.018
***ErbB2***	**0.234**	**0.044**
*ErbB3*	*0.584*	*0.054*
*ErbB4*	0.138	0.025
Int.	0.204	0.023

The average (mean) and standard deviation (SD) of branch lengths was calculated from 100 bootstrap replicates from real datasets. Bold indicates location of the root and italics indicate the longest branch in for gene, respectively.

A recently developed branch support measure, the approximate Likelihood Ratio Test (aLRT), can also be used to assess the support for branches [30]. This method compares the likelihood of a tree with the branch of interest collapsed to alternate model in which the branch has the length inferred from the data and tests whether there is sufficient data for the inferred branch to be “real”. Results from the Shimodaria-Hassegawa-like aLRT (a more conservative measure than the χ^2^-based aLRT) indicate there is strong support for each branch, particularly for post-duplication lineages (Figure 2). The more liberal χ^2^-based aLRT supported each branch with >0.95 values.

We also explicitly tested the location of the root for *Dlx*, *Col*, *Hox* and *ErbB* genes using parametric bootstrapping [31]. For each gene family we used the model of nucleotide substitution selected for the phylogenetic inference to simulate 100 replicate datasets on phylogenies with the alternate root, i.e. rooted at B for *Dlx/Col* and rooted at D for *Hox/ErbB*. These simulated datasets were then used to infer trees with ML, NJ and ME. If systematic biases in the data are responsible for the difference in rooting between *Dlx/Col* and *Hox/ErbB* then trees inferred from these simulated data should be incorrect (i.e. the root should be placed at some other internal branch). The correct tree, however, was inferred for all genes and methods in 94–98/100 replicate datasets, further indicating that these inference methods/data are robust to long-branch artifacts and systematic error/bias.

Finally, we tested alternate roots for *Dlx*, *Col*, *Hox* and *ErbB* genes using several methods implemented in the program CONSEL [32] that determine the confidence in the inferred tree by examining *P*-values for a set of alternate trees. The *P*-values of the Approximately Unbiased (AU) test [33], the Bootstrap Probability (NP) [32], the Bayesian Posterior Probability (PP) [32], the Kishino-Hasegawa (KH) test [34], the weighted Kishino-Hasegawa (wKH) test [32], the Shimodaira-Hasegawa (SH) test [35], and the Weighted Shimodaira-Hasegawa (wSH) test [32] were inferred for each possible rooting and in-group topology (15 alternate trees for *Col*, *Hox*, *and ErbB*; 3 alternate for *Dlx*). Like phylogenetic methods, these methods of selecting between competing phylogenetic tress each have strengths and weaknesses. For example, the AU and SH tests account for biases in selecting competing trees that are overlooked in the bootstrap probability and KH tests [33]. These corrections can lead to a more robustly supported tree, but can also make them too conservative [33]. By comparing the “best” tree scored by several methods (given as *P*-values), the effect of each methods assumptions on selecting the “best” tree can be ascertained. For each gene family we studied, the inferred root was significantly better than alternate rootings by most, if not all, of the selection methods indicating that method assumptions had little effect on picking the best tree (Tables 2–5).

**Table 2:** *Hox* cluster duplication topology tests.

Rank	Topology	−Ln	ΔlnL	AU	NP	PP	KH	wKH	SH	wSH
**1**	**(B(A(CD)))**	**−6448.62**		**0.93**	**0.757**	**0.946**	**0.854**	**0.854**	**0.986**	**0.984**
2	(B(C(AD)))	−6452.42	3.8	0.282	0.095	*0.021*	0.146	0.146	0.405	0.424
3	(B(D(CA)))	−6453.01	4.4	0.066	*0.011*	*0.012*	0.086	0.086	0.342	0.275
4	((AB)(CD))	−6453.27	4.6	0.121	0.076	*0.009*	0.115	0.115	0.315	0.297
5	(A(B(DC)))	−6453.27	4.6	0.120	0.076	*0.009*	0.115	0.115	0.315	0.297
6	((BC)(AD))	−6455.82	7.2	0.235	0.070	*0.001*	0.095	0.095	0.131	0.340
7	(C(B(AD)))	−6455.97	7.3	0.072	*0.010*	*0.001*	0.084	0.084	0.117	0.321
8	(A(D(BC)))	−6456.82	8.2	0.095	*0.023*	*3E-04*	0.076	0.076	0.096	0.258
9	(D(A(CB)))	−6456.82	8.2	0.096	*0.022*	*3E-04*	0.076	0.076	0.096	0.258
10	(C(D(BA)))	−6457.14	8.5	*0.019*	*0.001*	*2E-04*	*0.038*	*0.038*	0.06	0.171
**11**	**(D(C(AB)))**	**−6457.14**	**8.5**	***0.023***	***0.001***	***2E-04***	***0.038***	***0.038***	**0.06**	**0.189**
12	(C(A(DB)))	−6457.93	9.3	*0.015*	*0.001*	*9E-05*	*0.039*	*0.039*	*0.049*	0.213
13	(D(B(AC)))	−6457.95	9.3	*0.002*	*6E-05*	*8E-05*	*0.036*	*0.036*	*0.045*	0.192
14	((CA)(BD))	−6457.95	9.3	*0.002*	*6E-05*	*8E-05*	*0.036*	*0.036*	*0.045*	0.192
15	(A(C(DB)))	−6457.98	9.4	*0.002*	*1E-04*	*8E-05*	*0.038*	*0.038*	*0.047*	0.240

Tree topologies are ordered by decreasing likelihoods (shown as Rank).−Ln, negative log-likelihood of tree. ΔlnL, difference in likelihood score between this tree and tree rank 1. AU, the p-value of the approximately unbiased test. NP, bootstrap probability of the tree. PP, Bayesian posterior probability (calculated from BIC). KH, the p-value of the Kishino-Hasegawa test. wKS, the p-value of the weighted Kishino-Hasegawa test. SH, the p-value of the Shimodaira-Hasegawa test. wSH, the p-value of the weighted Shimodaira-Hasegawa test. The inferred tree is shown in bold and is ranked 1^st^, the competing tree is shown in bold ranked<2^nd^. Significant results are shown in italics (*P*>0.05).

**Table 3 pgen-1000349-t003:** *Col* cluster duplication topology tests.

Rank	Topology	−Ln	ΔlnL	AU	NP	PP	KH	wKH	SH	wSH
**1**	**(D(C(AB)))**	**−5444.85**		**0.89**	**0.594**	**0.984**	**0.754**	**0.968**	**0.754**	**0.969**
2	(D(A(BC)))	−5448.81	4.2	0.422	0.194	*0.014*	0.246	0.66	0.246	0.637
3	(D(B(AC)))	−5451.34	6.8	0.101	*0.020*	*0.001*	0.113	0.405	0.113	0.488
4	(C(D(AB)))	−5452.68	8.1	0.226	*0.049*	*3E-04*	0.122	0.443	0.122	0.401
5	((DC)(AB))	−5452.94	8.4	0.264	*0.044*	*2E-04*	0.113	0.42	0.113	0.378
6	(A(B(CD)))	−5455.68	11.1	0.163	*0.037*	*1E-05*	0.089	0.38	0.089	0.247
**7**	**(B(A(CD)))**	**−5455.75**	**11.2**	**0.104**	***0.011***	***1E-05***	**0.086**	**0.381**	**0.086**	**0.244**
8	(C(B(AD)))	−5456.12	11.5	0.159	*0.029*	*1E-05*	0.103	0.424	0.103	0.213
9	((AD)(BC))	−5456.62	12	0.119	*0.014*	*6E-06*	0.097	0.395	0.096	0.199
10	(C(A(BD)))	−5456.78	12.2	0.191	*0.025*	*5E-06*	0.093	0.398	0.093	0.193
11	(A(D(BC)))	−5457.17	12.6	0.028	*0.003*	*3E-06*	0.084	0.334	0.076	0.173
12	(A(C(BD)))	−5458.74	14.2	0.070	*0.004*	*7E-07*	0.066	0.353	0.066	0.123
13	((CA)(BD))	−5458.87	14.3	0.060	*0.002*	*6E-07*	0.064	0.353	0.064	0.119
14	(B(C(AD)))	−5460.14	15.6	*0.020*	*3E-04*	*2E-07*	*0.044*	0.262	*0.044*	*0.077*
15	(B(D(AC)))	−5461.66	17.1	*0.002*	*2E-05*	*4E-08*	*0.026*	0.187	*0.026*	*0.048*

Tree topologies are ordered by decreasing likelihoods (shown as Rank).−Ln, negative log-likelihood of tree. ΔlnL, difference in likelihood score between this tree and tree rank 1. AU, the p-value of the approximately unbiased test. NP, bootstrap probability of the tree. PP, Bayesian posterior probability (calculated from BIC). KH, the p-value of the Kishino-Hasegawa test. wKS, the p-value of the weighted Kishino-Hasegawa test. SH, the p-value of the Shimodaira-Hasegawa test. wSH, the p-value of the weighted Shimodaira-Hasegawa test. The inferred tree is shown in bold and is ranked 1^st^, the competing tree is shown in bold ranked<2^nd^. Significant results are shown in italics (*P*>0.05).

**Table 4 pgen-1000349-t004:** *ErbB* family cluster duplication topology tests.

Rank	Topology	−Ln	ΔlnL	AU	NP	PP	KH	wKH	SH	wSH
**1**	**(B(A(CD)))**	**−8450.73**		**0.75**	**0.596**	**0.878**	**0.683**	**0.683**	**0.925**	**0.945**
2	(A(B(CD)))	−8453.01	2.3	0.384	0.270	0.090	0.317	0.317	0.752	0.707
3	((CD)(AB))	−8454.07	3.3	0.299	0.123	*0.031*	0.233	0.233	0.728	0.653
**4**	**(D(C(AB)))**	**−8468.85**	**18.1**	***0.020***	***0.010***	***1E-08***	***0.036***	***0.027***	**0.131**	**0.074**
5	(C(D(AB)))	−8469.56	18.8	*0.003*	*5E-04*	*6E-09*	*0.027*	*0.018*	0.112	0.071
6	(B(D(AC)))	−8494.98	44.2	*3E-04*	*3E-05*	*5E-20*	*4E-04*	*4E-04*	*0.001*	*0.001*
7	(B(C(AD)))	−8494.98	44.2	*3E-04*	*3E-05*	*5E-20*	*4E-04*	*4E-04*	*0.001*	*0.001*
8	(A(D(BC)))	−8497	46.3	*1E-04*	*8E-06*	*7E-21*	*0.001*	*0.001*	*0.001*	*0.002*
9	(A(C(DB)))	−8497	46.3	*1E-04*	*8E-06*	*7E-21*	*0.001*	*0.001*	*0.001*	*0.002*
10	(D(B(AC)))	−8499.90	49.2	*4E-06*	*4E-06*	*4E-22*	*5E-04*	*5E-04*	*0.001*	*0.002*
11	(D(A(BC)))	−8499.91	49.2	*2E-06*	*4E-06*	*4E-22*	*5E-04*	*5E-04*	*0.001*	*0.003*
12	((CA)(BD))	−8502.19	51.5	*7E-68*	*3E-20*	*4E-23*	*2E-04*	*2E-04*	*2E-04*	*0.003*
13	(C(B(AD)))	−8502.22	51.5	*3E-36*	*1E-14*	*4E-23*	*2E-04*	*2E-04*	*2E-04*	*0.003*
14	((CB)(AD))	−8502.22	51.5	*3E-36*	*1E-14*	*4E-23*	*2E-04*	*2E-04*	*2E-04*	*0.003*
15	(C(A(BD)))	−8502.24	51.5	*4E-50*	*2E-17*	*4E-23*	*2E-04*	*2E-04*	*2E-04*	*0.004*

Tree topologies are ordered by decreasing likelihoods (shown as Rank).−Ln, negative log-likelihood of tree. ΔlnL, difference in likelihood score between this tree and tree rank 1. AU, the p-value of the approximately unbiased test. NP, bootstrap probability of the tree. PP, Bayesian posterior probability (calculated from BIC). KH, the p-value of the Kishino-Hasegawa test. wKS, the p-value of the weighted Kishino-Hasegawa test. SH, the p-value of the Shimodaira-Hasegawa test. wSH, the p-value of the weighted Shimodaira-Hasegawa test. The inferred tree is shown in bold and is ranked 1^st^, the competing tree is shown in bold ranked<2^nd^. Significant results are shown in italics (*P*>0.05).

**Table 5:** *Dlx* bi-gene cluster duplication topology tests.

Rank	Topology	−Ln	ΔlnL	AU	NP	PP	KH	wKH	SH	wSH
**1**	**(D(BA))**	**−4813.88**		**0.976**	**0.961**	**1**	**0.934**	**0.934**	**0.942**	**0.94**
**2**	**(B(AD))**	**−4823.76**	**9.9**	***0.041***	***0.032***	***5.00E-05***	**0.066**	**0.066**	**0.066**	**0.087**
3	(A(CD))	−4824.04	10.2	*0.021*	*0.009*	*4.00E-05*	*0.057*	*0.057*	*0.057*	0.117

Tree topologies are ordered by decreasing likelihoods (shown as Rank).−Ln, negative log-likelihood of tree. ΔlnL, difference in likelihood score between this tree and tree rank 1. AU, the p-value of the approximately unbiased test. NP, bootstrap probability of the tree. PP, Bayesian posterior probability (calculated from BIC). KH, the p-value of the Kishino-Hasegawa test. wKS, the p-value of the weighted Kishino-Hasegawa test. SH, the p-value of the Shimodaira-Hasegawa test. wSH, the p-value of the weighted Shimodaira-Hasegawa test. The inferred tree is shown in bold and is ranked 1^st^, the competing tree is shown in bold ranked<2^nd^. Significant results are shown in italics (*P*>0.05).

## Discussion

Nearly 40 years after Ohno first proposed that the vertebrate genome evolved through two successive rounds of whole genome duplication [1], the role of large-scale gene, chromosome and/or whole genome duplications in vertebrate genome evolution remains controversial. While the exact mechanisms of genome expansion are debated, there is now little doubt that expansion occurred. Analysis of human paralogs, for example, indicates that both large- and small-scale duplications played an important role in vertebrate genome evolution, with many of the duplications occurring in large blocks (*en bloc*) of chromosomes or chromosome segments [2],[8]. These duplication events occurred in at least three waves, the largest of which occurred in the early stages of vertebrate evolution coincident with expectations of the 2R model [8].

The *Hox* clusters have played a central role in the genome duplication story, largely because they conform to the 1∶4 expectation of the 2R hypothesis and are tightly linked to each other and several non-Hox genes. However, numerous studies of the duplications of the Hox clusters and linked genes have failed to reach a consensus on the mechanisms, number and order of duplications [2]–[16], [21]–[26],[36]. Many of these studies were hampered by limited sequence data and poor taxon sampling, lack of appropriate out-group data or computational limitations that prevented the use of computationally intensive methods of phylogenetic inference (such as Bayesian inference and maximum likelihood). Given these difficulties it is not surprising that nearly every study found support for a different duplication order.

Our analyses of the *Dlx*, *Col*, *Hox* cluster, and *ErbB* gene duplication histories identified an unexpected pattern that divides the core paralogon into two clear topological regions: the *Dlx/Col* region supporting a (D(C(B,A))) branching order while the *Hox/ErbB* region supports an alternate rooting of (B(A(C,D))). The topology of each region is moderately- to highly-supported by nonparametric bootstrap support values from multiple methods of phylogenetic inference (ML, NJ, ME) and highly supported by Bayesian posterior probabilities; this congruence of topologies among methods can itself be taken as a strong indicator of tree accuracy [37].

Trees that differ only in the placement of the root are generally thought to arise because of out-group misplacement either from LBA artifacts, other kinds of systemic bias or short internal branch lengths [28],[29]. Our numerous tests, however, indicate that root misplacement is not likely to be the cause of the two different topologies found for genes in the *Hox* paralogon. Indeed, these topologies appear to be particularly robust to the kind of systemic error and bias that would cause out-group misplacement. Thus, we conclude that the split in duplication history is likely to be real and results from two chromosomal rearrangements that occurred between the *Col* genes and *Hox* clusters after the duplication events but before the radiation of extant vertebrates.

Interestingly, our finding of structural changes in the *Hox* paralogon bearing chromosomes following the ancestral vertebrate genome duplication and recombination breakpoints between human Hox paralogon members (see below) may shed light on previous findings of differential molecular evolution in anterior (3′) and posterior (5′) *Hox* genes [38],[39]. Several studies have shown that the rate of molecular evolution is not uniformly distributed across the genome, with genes evolving faster near genomic regions with high recombination rates than genes near regions with low recombination rates [40],[41]. The findings that posterior *Hox* genes evolve faster than anterior and middle Hox genes within gnathostomes (termed *Laxitas terminalis*) [38], between phyla and subphyla (termed Posterior Flexibility) [39] and after genome duplications [42] may reflect this general trend.

Furlong and Holland [18] have argued that asymmetrical trees and incongruent topologies between linked genes are not evidence against whole genome duplications as some have argued [11]–[16],[43], but are in fact a prediction of the 2R-model if both duplications occurred by rapid autotetraploidy. For example, if the diploidization after the first genome duplication (tetraploidization) was nearly complete by the time of the second duplication event, then gene trees would be sequential such as (A(B(CD))); during this pseudo-octaploid phase crossovers are likely to occur, because sequence divergence between homologous regions is still relatively low, resulting in gene trees that are incongruent between linked genes. However, given that sequence similarity is low enough to allow recombination, how is it possible to have relatively strong phylogenetic signal? The answer to this paradox likely lies in the evolution of Hox genes after duplications. For example, positive selection acting on Hox genes after the cluster duplications in teleost fish actually generated strong phylogenetic signal by rapidly fixing amino acid substitutions that preserved information on the duplication history [44]. Simiarly, positive selection acted on the *Hox* genes immediately after cluster duplications in vertebrates, rapidly fixing amino acid substitutions [42] and likely preserving a phylogenetic footprint of duplication order.

**Figure 4:**
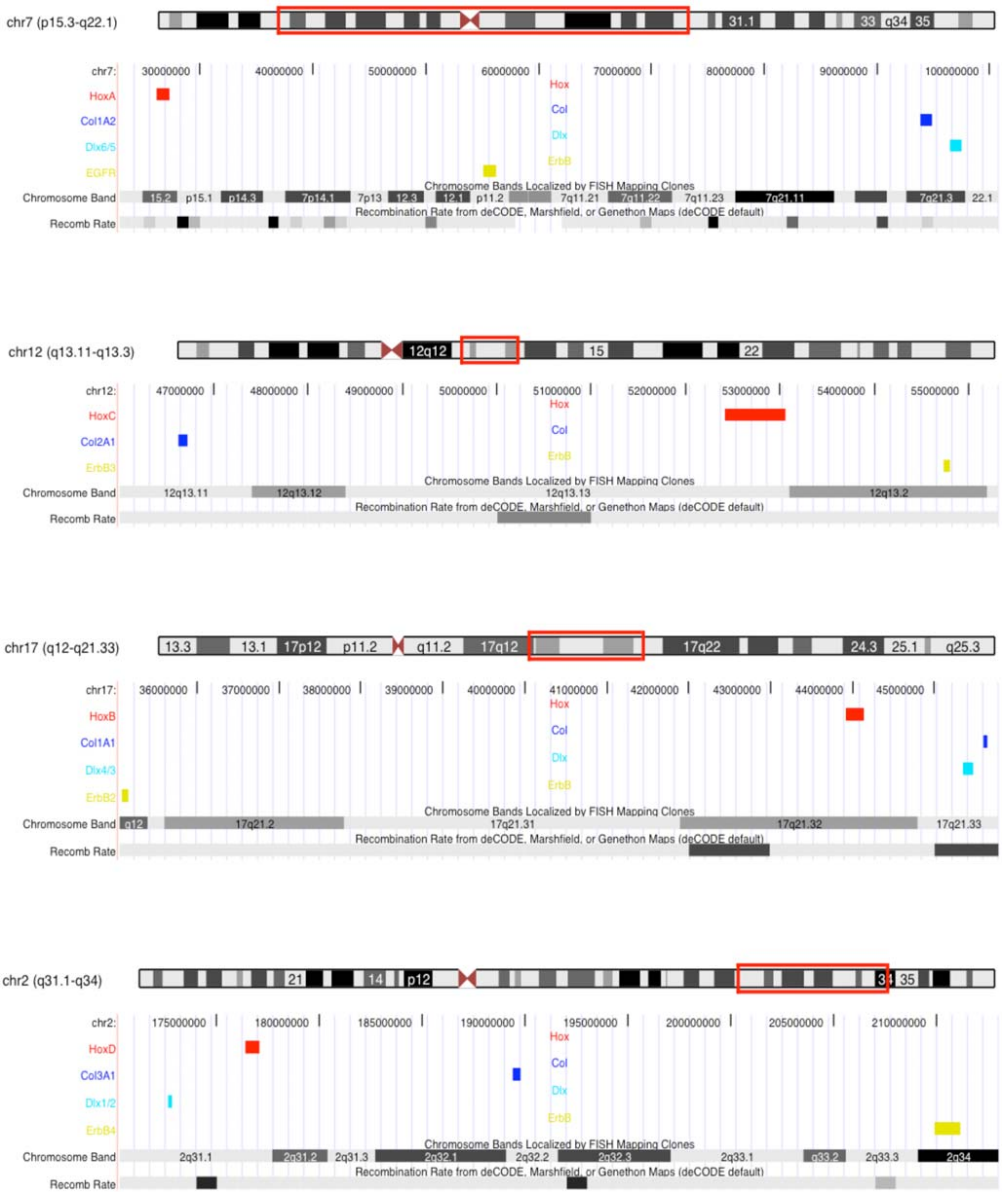
Recombination rate variation across human Hox paralogon containing chromosomes. The location of the *Hox* paralogon on human chromosome ideograms (human Chr7, Chr12, Chr17, Chr2) are boxed in red. *Hox* clusters are colored red, *Col* genes blue, *Dlx* genes light blue and *ErbB* genes green. The region below the ideograms corresponds to that region of the chromosome. Chromosomal bands are shown, as are the deCODE sex average recombination rate (Recomb Rate track) and repetitive element tracks. Darker gray bands in recombination rate track indicate higher than average recombination rates. Data from the UCSC genome browswer.

Our data suggest that at least two chromosomal crossover events occurred between the vertebrate protochromosomes bearing the core *Hox* paralogon genes, but are such chromosomal rearrangements likely? Several studies have shown that large- and small-scale chromosomal rearrangements are common after whole genome duplications [45]–[48], indicating that rearrangement of the vertebrate protochromosomes was extremely likely. For example, chromosomal rearrangements occurred within a few generations of hybridization in allotetraploid crosses of *Arabidopsis suecica* and *Arabidopsis thaliana* [47] and are common in autotetraploid Salmonid fish [49]. Inferring the pattern of chromosomal rearrangement after the vertebrate genome duplications may not be possible at a fine scale, but clues to the frequency of crossover events involving the core Hox paralogon genes can be found in a recent map of recombination rates in the human genome [50]. Remarkably, several windows of high recombination rate are found between genes in the paralogon (Figure 4). While these regions of high recombination rate indicate that crossovers occur between homologous chromosomes bearing the *Hox* paralogon in humans, they can only suggest that similar processes were at work after the whole genome duplications in the vertebrate ancestor.

### Conclusions

**Figure 5:**
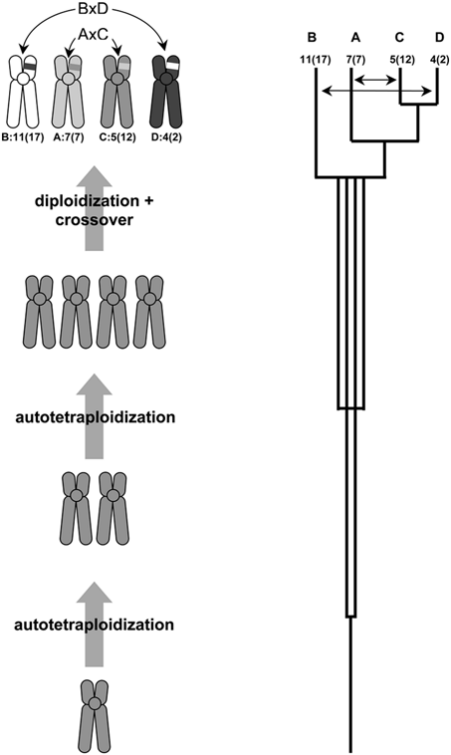
Reconstruction of the Hox cluster duplication history. If genome duplications occur in close succession, diploidization will be sequential from an octoploid or pseudo-octoploid state. Gene trees will then reflect the order of diploidization of chromosomes, rather than the order of chromosome duplication and the tree topology will be sequential (after Furlong and Holland 2001). Resolving the incongruent gene tree topologies for *Dlx/Col* and *Hox/ErbB* genes requires two chromosomal rearrangements between chromosomes carrying the *HoxB* and *D* clusters (*BxD*) and *HoxA* and *C* clusters (*AxC*) in the intergenic region between the *collagen* genes and the *Hox13* paralogs. Chromosomes are labeled with respect to *Hox* cluster (*A–D*), the chromosomal location of that *Hox* cluster in the human genome and the chromosomal location in the vertebrate ancestor (ancestral karyotype information from (Kohn et al., 2006); shown as *Hox* cluster: ancestral vertebrate chromosome (human chromosome). Double-sided arrows indicate crossover events.

The pattern of gene duplications for the core *Hox* paralogon genes is best explained by the proposal of Furlong and Holland [18] and provides a convincing case of a chromosomal crossover event between vertebrate protochromosomes 11 and 4, and 7 and 5 over 450 MYA (Figure 5)[51]. Most importantly, the identification of these chromosomal rearrangements in a highly conserved vertebrate syntenic block reconciles the conflicting interpretations of gene trees for this region and supports the hypothesis that large scale chromosomal or whole genome duplications contributed to vertebrate genome evolution. Further, these results support the proposal of Furlong and Holland [18] that the duplication events were the result of autotetraploidy, and that vertebrates are pseudo-octaploids.

## Materials and Methods

### Phylogenetic Analysis

Genes for *Dlx*, *Collagen (Col)*, *Hox* homeodomains, and *ErbB* were downloaded from GenBank or identified from BLAST searches of nucleotide and amino acid databases; the alignments are available from VJL by request. The homeodomains of all paralog members for each Hox cluster were concatenated into a single alignment. Amino acid sequences for all genes were aligned with MUSCLE [52],[53] and adjusted by eye. Regions with large gaps, ambiguous alignment or repetitive sequences were removed from all genes.

Appropriate models of sequence evolution were estimated for each dataset with the program ModelGenerator [54] with the gamma rate parameter (approximated with 4 rate categories) and the proportion of invariable sites estimated from the data where appropriate. Phylogenetic trees were reconstructed using neighbor-joining (NJ), distance (minimum evolution), and maximum likelihood (ML) algorithms implemented in the PHYLIP v3.6 package of programs. ML trees were also generated using PhyML v2.4 [55]. (There were no significant differences between these implantations of ML and results from PhyML are reported.) Branch support was assessed with 1000 bootstrap resamplings for NJ, distance, and ML. The approximate likelihood ratio test implemented in PhyML was also used to infer branch support for ML trees. Bayesian trees were generated with Mr.Bayes v3.0 [56], running 2 sets of 4 chains for 10,000,000 generations sampling every 1000^th^ tree. Convergence of model parameters was assayed using Tracer (http://tree.bio.ed.ac.uk/software/tracer/) and ensuring the average standard deviation of split frequencies was less than 0.01.

### Tests of Tree Topologies

The 15 alternate rootings of *Hox*, *Collagen* and *ErbB* genes, and the 3 alternate rootings of the *Dlx* genes were directly tested to determine if the roots inferred from phylogenetic analyses were significantly better than all alternate roots using the methods implemented in the program CONSEL [32]. Parametric bootstrap tests were also used to test for the effects of rooting of *Collagen/Dlx* at *HoxB* (these genes are inferred to be rooted at *HoxD*) and *Hox/ErbB* at *HoxD* (these genes are inferred to be rooted at HoxB). 100 replicate datasets were generated using Seq-Gen (http://tree.bio.ed.ac.uk/software/seqgen/) for each gene family using a model of evolution that matched the model inferred from the real dataset and a tree topology that changed the location of the root. Similarly, to test for the affect of branch lengths on the inferred tree topology, 100 replicate datasets were generated using Seq-Gen for each branch length set using a model of evolution (similar to that inferred for the Hox gene dataset). Internal branch lengths in the model tree (from which Seq-Gen generated simulated datasets) were incrementally increased from 0–2. Finally, the effect of root misplacement and branch-length on the accuracy of tree inferences was examined by inferring trees from each replicate dataset with ML and counting the frequency of that the true tree was inferred or plotting the branch length against the average internal branch support (shown in Figure 3).

## References

[1] Ohno S (1970). Evolution by gene duplication.

[2] Larhammar D, Lundin LG, Hallbook F (2002). The human *Hox*-bearing chromosome regions did arise by block or chromosome (or even genome) duplications.. Genome Res.

[3] Lundin LG (1993). Evolution of the vertebrate genome as reflected in paralogous chromosomal regions in man and the house mouse.. Genomics.

[4] Meyer A, Schartl M (1999). Gene and genome duplications in vertebrates: the one-to-four (-to-eight in fish) rule and the evolution of novel gene functions.. Curr Opin Cell Biol.

[5] Spring J (1997). Vertebrate evolution by interspecific hybridization—are we polyploid?. FEBS Lett.

[6] Wang Y, Gu X (2000). Evolutionary patterns of gene families generated in the early stage of vertebrates.. J Mol Evol.

[7] Dehal P, Boore JL (2005). Two Rounds of Whole Genome Duplication in the Ancestral Vertebrate.. PLoS Bio.

[8] Gu X, Wang Y, Gu J (2002). Age distribution of human gene families shows significant roles of both large- and small-scale duplications in vertebrate evolution.. Nat Genet.

[9] Guigo R, Muchnik I, Smith TF (1996). Reconstruction of ancient molecular phylogeny.. Mol Phylogenet and Evol.

[10] McLysaght A, Hokamp K, Wolfe KH (2002). Extensive genomic duplication during early chordate evolution.. Nat Genet.

[11] Friedman R, Hughes AL (2001). Pattern and timing of gene duplication in animal genomes.. Genome Res.

[12] Friedman R, Hughes AL (2003). The temporal distribution of gene duplication events in a set of highly conserved human gene families.. Mol Biol and Evol.

[13] Hughes AL, Friedman R (2003). 2R or not 2R: testing hypotheses of genome duplication in early vertebrates.. J Struct Funct Genomics.

[14] Hughes AL (1999). Phylogenies of developmentally important proteins do not support the hypothesis of two rounds of genome duplication early in vertebrate history.. J Mol Evol.

[15] Hughes AL (1998). Molecular Phylogenetic tests of the hypothesis of block duplication of homologous genes on human chromosomes 6, 9, and 1.. Mol Biol and Evol.

[16] Hughes AL, da Silva J, Friedman R (2001). Ancient genome duplications did not structure the human Hox-bearing chromosomes.. Genome Res.

[17] Chowdhary BP, Raudsepp T, Frönicke L, Sherthan H (1998). Emerging patterns of comparative genome organization in some mammalian species as revealed by zoo-FISH.. Genome Res.

[18] Gregory SG, Sekhon M, Schein J, Zhao S, Osoegawa K (2002). A physical map of the mouse genome.. Nature.

[19] Murphy WJ, Stanyon R, O'Brien SJ (2002). Evolution of mammalian genome organization inferred from comparative gene mapping.. Genome Res.

[20] Wagner GP, Amemiya C, Ruddle F (2003). Hox cluster duplications and the opportunity for evolutionary novelties.. Proc Natl Acad Sci U S A.

[21] Furlong R, Holland PW (2002). Were vertebrates octoploid?. Philos Trans R Soc Lond B Biol Sci.

[22] Kappen C, Ruddle FH (1993). Evolution of a regulatory gene family: HOM/HOX genes.. Curr Opin Genet Dev.

[23] Zhang J, Nei M (1996). Evolution of Antennapedia-Class Homeobox Genes.. Genetics.

[24] Bailey WJ, Kim J, Wagner GP, Ruddle FH (1997). Phylogenetic reconstruction of vertebrate Hox cluster duplications.. Mol Biol and Evol.

[25] Stock DW (2005). The Dlx gene complement of the leopard shark, Triakis semifasciata, resembles that of mammals: implications for genomic and morphological evolution of jawed vertebrates.. Genetics.

[26] Neidert AH, Virupannavar V, Hooker W, Langeland JA (2001). Lamprey Dlx genes and early vertebrate evolution.. Proc Natl Acad Sci U S A.

[27] Holder M, Lewis PO (2003). Phylogeny estimation: Traditional and Bayesian approaches.. Nat Rev Genet.

[28] Holland BR, Penny D, Hendy MD (2003). Outgroup misplacement and phylogenetic inaccuracy under a molecular clock–a simulation study.. Syst Biol.

[29] Bergsten J (2005). A review of long-branch attraction.. Cladistics.

[30] Anisimova M, Gascuel O (2006). Approximate likelihood-ratio test for branches: A fast, accurate, and powerful alternative.. Syst Biol.

[31] Goldman N, Anderson JP, Rodrigo AG (2000). Likelihodd-based tests of topologies in phylogenetics.. Syst Biol.

[32] Shimodaira H, Hasegawa M (2001). CONSEL: for assessing the confidence of phylogenetic tree selection.. Bioinformatics.

[33] Shimodaira H (2002). An approximately unbiased test of phylogenetic tree selection.. Syst Biol.

[34] Kishino H, Hasegawa M (1989). Evaluation of the maximum likelihood estimate of the evolutionary tree topologies from DNA sequence data, and the branching order in hominoidea.. J Mol Evol.

[35] Shimodaira H, Hasegawa M (1999). Multiple comparisons of log-likelihoods with applications to phylogenetic inference.. Mol Biol Evol.

[36] Popovici C, Leveugle M, Birnbaum D, Coulier F (2001). Homeobox gene clusters and the human paralogy map.. FEBS Lett.

[37] Kim J (1998). Large-scale phylogenies and measuring the performance of phylogenetic estimators.. Syst Biol.

[38] Ferrier DE, Minguillon C, Holland PW, Garcia-Fernandez J (2000). The amphioxus Hox cluster: deuterstome posterior flexability and Hox14.. Evol Dev.

[39] van der Hoeven F, Sordino P, Fraudeau N, Izisua-Belmonte JC, Duboule D (1996). Telesost HoxD and HoxA genes: comparison wiht tetrapods and functional evolution of the HoxD complex.. Mech Dev.

[40] Evans AL, Mena PA, McAllister BF (2007). Positive selection near an inversion breakpoint on the neo-X chromosome in Drosophila americana.. Genetics In Press.

[41] Marques-Bonet T, Navarro A (2005). Chromosomal rearrangements are associated with higher rates of molecular evolution in mammals.. Gene.

[42] Lynch V, Roth J, Wagner G (2006). Adaptive evolution of Hox-gene homeodomains after cluster duplications.. BMC Evol Biol.

[43] Martin A (2001). Is Tetralogy True? Lack of Support for the “One-to-Four Rule”.. Mol Biol and Evol.

[44] Crow KD, Stadler PF, Lynch VJ, Amemiya C, Wagner GP (2006). The “Fish-Specific” Hox Cluster Duplication Is Coincident with the Origin of Teleosts.. Mol Biol Evol.

[45] Hufton AL, Groth D, Vingron M, Lehrach H, Poustka AJ (2008). Early vertebrate whole genome duplications were predated by a period of intense genome rearrangement.. Genome Res.

[46] Nakatani Y, Takeda H, Kohara Y, Morishita S (2007). Reconstruction of the vertebrate ancestral genome reveals dynamic genome reorganization in early vertebrates.. Genome Res.

[47] Pontes O, Neves N, Silva M, Lewis MS, Madlung A (2004). Chromosomal locus rearrangements are a rapid response to formation of the alllotetraplid Aradidopsis suecica genome.. Proc Natl Acad Sci U S A.

[48] Sémon M, Wolfe KH (2007). Consequences of genome duplication.. Curr Opin Genet Dev.

[49] Phillips R, Ráb P (2001). Chromosome evolution in the Salmonidae (Pisces): an update.. Bio Reivews.

[50] McVean GAT, Myers SR, Hunt S, Deloukas P, Bentley DR (2004). The fine scale structure of recombination rate variation in the human genome.. Science.

[51] Kohn M, Hogel J, Vogel W, Minich P, Kehrer-Sawatzki H (2006). Reconstruction of a 450-My-old ancestral vertebrate protokaryotype.. Trends Genet.

[52] Edgar RC (2004). MUSCLE: multiple sequence alignment with high accuracy and high throughput.. Nucleic Acids Res.

[53] Edgar RC (2004). MUSCLE: a multiple sequence alignment method with reduced time and space complexity.. BMC Bioinformatics.

[54] Keane T, Creevey C, Pentony M, Naughton T, McLnerney J (2006). Assessment of methods for amino acid matrix selection and their use on empirical data shows that ad hoc assumptions for choice of matrix are not justified.. BMC Evol Biol.

[55] Guindon S, Gascuel O (2003). A simple, fast, and accurate algorithm to estimate large phylogenies by maximum likelihood.. Sys Biol.

[56] Huelsenbeck JP, Ronquist F (2001). MRBAYES: Bayesian inference of phylogenetic trees.. Bioinformatics.

